# In vitro reversion of activated primary human hepatic stellate cells

**DOI:** 10.1186/s13069-015-0031-z

**Published:** 2015-08-06

**Authors:** Adil El Taghdouini, Mustapha Najimi, Pau Sancho-Bru, Etienne Sokal, Leo A. van Grunsven

**Affiliations:** Liver Cell Biology Lab, Vrije Universiteit Brussel (VUB), Laarbeeklaan 103, 1090 Brussels, Belgium; Laboratory of Pediatric Hepatology and Cell Therapy, Institut de Recherche Expérimentale et Clinique (IREC), Université Catholique de Louvain, Brussels, Belgium; Institut d’Investigacions Biomèdiques August Pi i Sunyer (IDIBAPS), Barcelona, Spain

**Keywords:** Fibrosis, Hepatic stellate cells, Reversion, Quiescent, Inactivation, Gene expression profiling

## Abstract

**Background:**

Liver fibrosis is characterized by the excessive formation and accumulation of matrix proteins as a result of wound healing in the liver. A main event during fibrogenesis is the activation of the liver resident quiescent hepatic stellate cell (qHSC). Recent studies suggest that reversion of the activated HSC (aHSC) phenotype into a quiescent-like phenotype could be a major cellular mechanism underlying fibrosis regression in the liver, thereby offering new therapeutic perspectives for the treatment of liver fibrosis. Whether human HSCs have the ability to undergo a similar reversion in phenotype is currently unknown. The aim of the present study is to identify experimental conditions that can revert the in vitro activated phenotype of primary human HSCs and consequently to map the molecular events associated with this reversion process by gene expression profiling.

**Results:**

We find that epidermal growth factor (EGF) and fibroblast growth factor 2 (FGF2) synergistically downregulate the expression of *ACTA2* and *LOX* in primary human aHSCs. Their combination with oleic acid, palmitic acid, and retinol further potentiates a more quiescent-like phenotype as demonstrated by the abundant presence of retinyl ester-positive intra-cytoplasmic lipid droplets, low expression levels of activation markers, and a reduced basal as well as cytokine-stimulated proliferation and matrix metalloproteinase activity. Gene expression profiling experiments reveal that these in vitro reverted primary human HSCs (rHSCs) display an intermediary phenotype that is distinct from qHSCs and aHSCs. Interestingly, this intermediary phenotype is characterized by the increased expression of several previously identified signature genes of in vivo inactivated mouse HSCs such as *CXCL1*, *CXCL2*, and *CTSS*, suggesting also a potential role for these genes in promoting a quiescent-like phenotype in human HSCs.

**Conclusions:**

We provide evidence for the ability of human primary aHSCs to revert in vitro to a transitional state through synergistic action of EGF, FGF2, dietary fatty acids and retinol, and provide a first phenotypic and genomic characterization of human in vitro rHSCs.

**Electronic supplementary material:**

The online version of this article (doi:10.1186/s13069-015-0031-z) contains supplementary material, which is available to authorized users.

## Background

In response to injury, tissue can heal and restore its normal function through a controlled sequence of events known as wound healing. However, when the insult is of chronic nature, it can impair the wound healing response and subsequently develop into tissue fibrosis, a condition characterized by the excessive accumulation of matrix proteins and associated with severe morbidity and mortality [1]. Different cellular sources, including tissue-specific fibroblasts, bone marrow-derived progenitor cells, pericytes, and epithelial cells, have been suggested to give rise to myofibroblasts, the major source of extracellular matrix (ECM) components in the fibrotic organ [2]. In the liver, however, the resident hepatic stellate cell (HSC) has unambiguously been identified as the predominant source of myofibroblasts, irrespective of the underlying disease etiology [3].

In the normal liver, quiescent HSCs (qHSCs) reside in a virtual space (of Disse) between the hepatocytes and liver sinusoidal endothelial cells and are characterized by the abundance of cytoplasmic lipid droplets containing up to 80 % of the total vitamin A body reserve [4]. Besides their well-known role in the regulation of retinoid and ECM homeostasis, there is evidence that HSCs can regulate the sinusoidal blood flow and stimulate angiogenesis [5, 6]. Following chronic liver injury, qHSCs undergo a process of activation, during which they transdifferentiate into cells with a fibrogenic, myofibroblast-like phenotype characterized by the loss of vitamin A-containing lipid droplets, increased smooth muscle actin (*ACTA2*) expression, and an augmentation in ECM production and secretion [4, 7].

Initially, this activation process was considered to be unidirectional while the principal ability of the fibrotic liver to revert to a normal state upon cessation of injury [8] was mainly attributed to the clearance of activated HSCs (aHSCs) that undergo apoptosis [9, 10]. However, different studies strongly imply that at least in rodents, the activated phenotype of HSCs can be modulated and reverted to a quiescent-like state, both in vitro [11, 12] and in vivo [13, 14]. The identification of aHSCs as the main source of ECM in the fibrotic liver in conjunction with recent work showing that HSC inactivation could drive fibrosis regression opens new therapeutic perspectives. Indeed, identifying and understanding the molecular events governing the reversion of an aHSC will be helpful in efficiently targeting liver fibrosis. Although convincingly shown in rodent HSCs, inactivating conditions for human primary HSCs have barely been investigated. In the present study, we aimed at identifying experimental conditions that can induce a reversion of the activated human HSC phenotype and at mapping the molecular events associated with this process.

We find that epidermal growth factor (EGF) and fibroblast growth factor 2 (FGF2) act synergistically to downregulate the expression of *ACTA2* and lysyl oxidase (*LOX*) in primary human HSCs. The combined effect is further potentiated by dietary fatty acids and retinol to reach levels of expression that are similar to those measured in freshly isolated, non-cultured human qHSCs. Further, this cocktail induces a quiescent-like phenotype as demonstrated by the acquisition of retinyl ester-containing cytoplasmic lipid droplets and a low proliferation rate. Gene expression profiling of human primary qHSCs and in vitro aHSCs and reverted HSCs (rHSCs) reveals many additional pro-fibrogenic genes to be downregulated in rHSCs. However, despite their phenotype, the overall expression profile of rHSCs is not similar to that of qHSCs and differs from that of aHSCs, but shows increased expression of previously identified in vivo inactivated mouse HSC (iaHSC) signature genes [13, 15].

## Results

### EGF and FGF2 act synergistically with dietary fatty acids and retinol to revert the culture activated phenotype of human HSCs

**Fig. 1:**
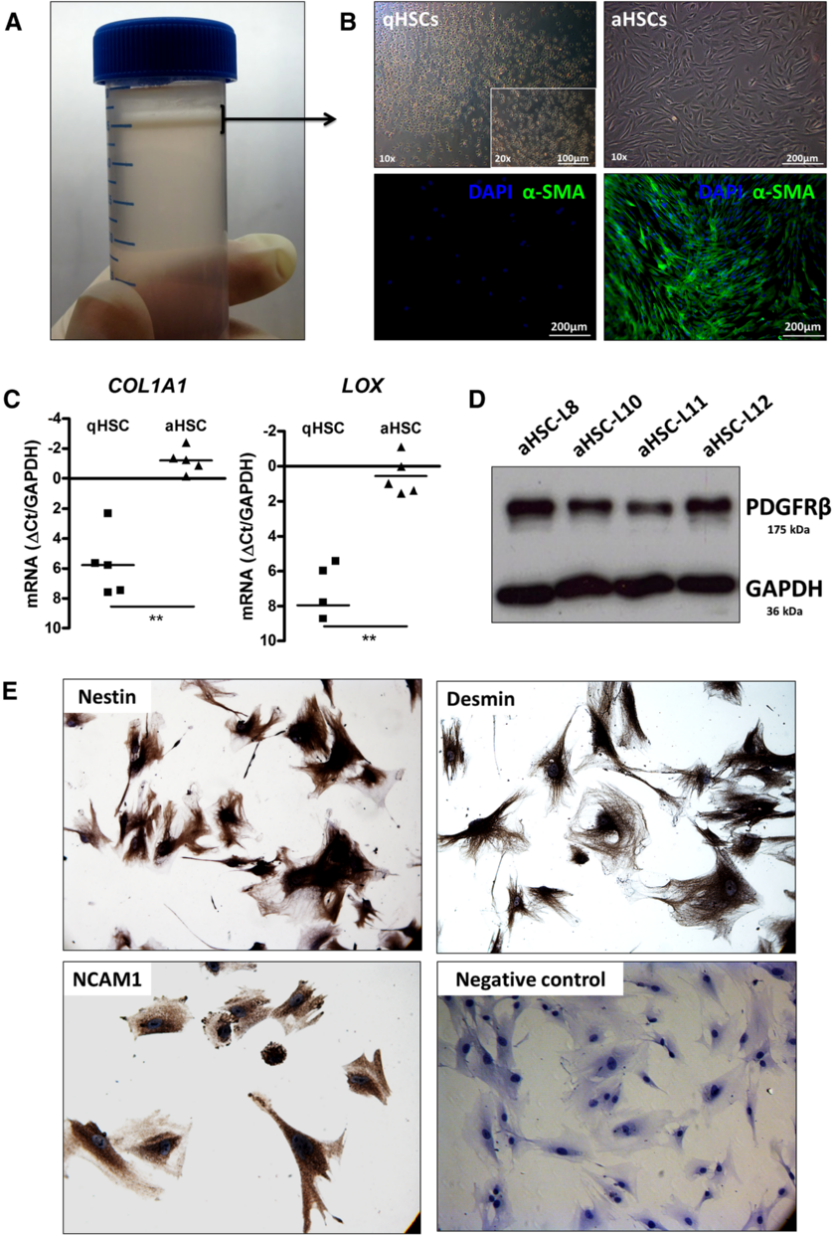
Isolation and culture activation of human primary HSCs. **a** Quiescent HSC-enriched cell layer (*arrow*) following an 8 % Nycodenz density gradient centrifugation. **b** Light microscopic and α-SMA immunocytochemistry images of the freshly isolated, qHSC-enriched cell population and fully culture activated HSCs (passage 4). **c** Log2 mRNA expression levels of *COL1A1* and *LOX* in freshly isolated, non-plated qHSCs and aHSCs from five different donors. In the graphs, **p < 0.01. **d** PDGFRβ and GAPDH protein levels in aHSCs from four donors. **e** Positive immunostainings for the neural markers NCAM1, nestin, and desmin

Human HSCs are isolated by density gradient centrifugation from the non-parenchymal cell fraction of healthy liver donors (Table 1) and seeded on plastic culture dishes to induce activation (Fig. 1a, b). The activated status of the cells is assessed by their typical myofibroblastic phenotype, the strong increase in alpha smooth muscle actin (α-SMA) protein by immunocytochemistry (Fig. 1b) and collagen type 1 alpha 1 (*COL1A1)* and lysyl oxidase (*LOX*) expression by RT-qPCR (Fig. 1c). The aHSCs express high protein levels of platelet-derived growth factor receptor beta (PDGFRβ), a membrane receptor associated with HSCs (Fig. 1d). Moreover, the cells stain positive for different markers previously shown to be expressed by HSCs, i.e., NCAM1, nestin, and desmin [16–19] (Fig. 1e).

**Fig. 2:**
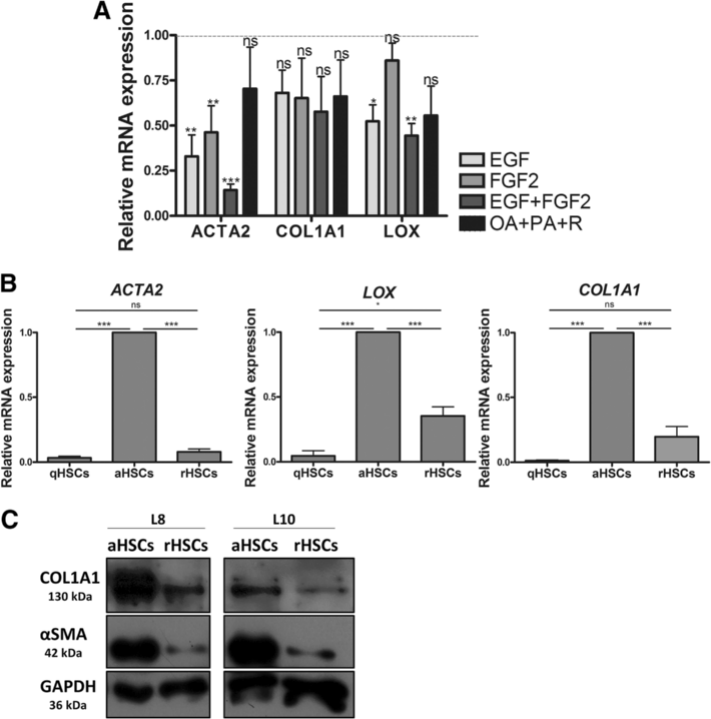
EGF, FGF2, retinol, palmitic acid, and oleic acid act synergistically to negatively regulate the expression of *ACTA2*, *COL1A1*, and *LOX* in human primary HSCs. **a** Human aHSCs were exposed to recombinant human EGF (20 ng/mL), FGF2 (10 ng/mL), a combination of both, or a combination of oleic acid (100 μM) (OA), palmitic acid (100 μM) (PA), and retinol (5 μM) for 5 days. Extracted RNA was processed and analyzed for *ACTA2*, *COL1A1*, and *LOX* expression by RTq-PCR. Results are presented as relative fold change to untreated control cells (*dotted line*). **b** mRNA expression levels of *ACTA2*, *COL1A1*, and *LOX* in non-cultured quiescent (q), culture activated (a), and reverted (r) HSCs incubated for 5 days with RM (20 ng/mL EGF, 10 ng/mL FGF2, 100 μM OA, 100 μM PA, 5 μM R) from three different (corresponding) donors. The expression levels are presented as relative fold change to aHSCs. The results presented are from three to five different donors. In the graphs, the results are displayed as means ± SEM. *ns* not significant, *p* ≥ 0.05, *p < 0.05, **p < 0.01, ***p < 0.001. **c** α-SMA, COL1A1, and GAPDH protein levels in aHSCs and rHSCs from corresponding donors

**Fig. 3:**
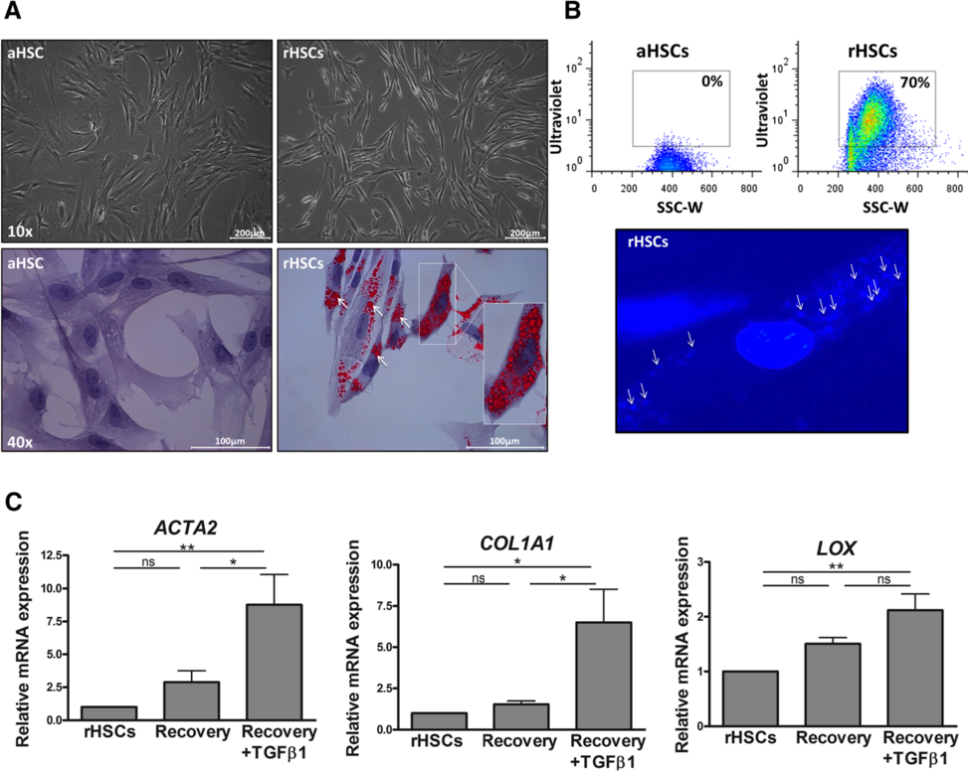
EGF, FGF2, retinol, palmitic acid, and oleic acid restore a reversible, quiescent-like phenotype in culture activated human primary HSCs. **a** Light microscopic and Oil Red O staining images of aHSCs and rHSCs. **b** FACS detection and microscopic image of the intrinsic fluorescence (at a wavelength of ~328 nm) of all-trans retinyl esters in UV-excited aHSCs and rHSCs. The *arrows* highlight the intrinsic fluorescent signal of retinyl esters inside the cytoplasmic lipid droplets. **c** The reversibility of the RM-induced downregulation of *ACTA2*, *COL1A1*, and *LOX* was assessed by washing the cells after a 5-day incubation period, followed by an additional 2-day culture in the presence or absence of recombinant human TGFβ (10 ng/mL), in FBS-free medium. The expression levels are presented as relative fold change to rHSCs. The results presented are from three to five different donors. In the graphs, the results are displayed as means ± SEM. *ns* not significant, *p* ≥ 0.05, *p < 0.05, **p < 0.01

We have tested different culture conditions for their ability to revert the activated phenotype of HSCs. These conditions include pharmacological agents previously shown to inhibit rodent HSC activation (i.e., TWS119, valproic acid (VPA), valinomycin, carbonyl cyanide-*p*-trifluoromethoxyphenylhydrazone (FCCP), tin-protoporphyrin IX dichloride (SnPP), bafilomycin (BFM), hydroxychloroquine (HCQ), 3-methyladenine (3-MA)) (Additional file 1: Figure S1A), an adipogenic differentiation mixture (MDI) [12] (Additional file 1: Figure S1B), and a diverse pool of growth factors and cytokines (data not shown). While our experiments reveal that these agents merely induce cell death (Valinomycin, BFM, 3-MA) in human HSCs or show limited efficacy to inactivate the cells especially at the level of *COL1A1* expression (TWS119, VPA, FCCP, SnPP, HCQ, MDI), they also reveal that FGF2 and EGF significantly downregulate the expression of *ACTA2* or *ACTA2* and *LOX*, respectively (Fig. 2a). We further find that the combined effect of EGF and FGF2 on the expression of *ACTA2*, *COL1A1*, and *LOX* expression is strongly potentiated by a mixture of dietary fatty acids (oleic acid (OA); palmitic acid (PA)) and retinol (R) to reach levels of expression that are similar to those measured in freshly isolated, non-cultured qHSCs (Fig. 2b), with decreased levels of *ACTA2* and *COL1A1* confirmed at the protein level (Fig. 2c). Of note, the mixture of dietary components alone has no significant effect on activation (Fig. 2a). HSCs reverted by this mix of growth factors and dietary components (further referred to as reverting medium (RM)) display a quiescent-like phenotype, characterized by a thinner cell body and the presence of intra-cytoplasmic lipid droplets (Fig. 3a). Although HSCs have strongly reduced expression levels of activation genes as soon as 2 days after incubation with RM, intra-cytoplasmic lipid droplet accumulation is only observable after 4 days (Additional file 1: Figure S2). Therefore, functional analysis and gene expression profiling were performed on cells that were incubated with RM for at least 4 days.

**Table 1:** Clinical characteristics of the HSC donors

Donor	Healthy status	Age	Gender	Ischemia time
L4	Healthy	12 years	Female	16 h 30 min
L8	Healthy	1 day	Male	4 h 40 min
L10	Healthy	7 months	Female	5 h 20 min
L11	Healthy	7 days	Male	4 h 25 min
L12	FH	13 years	Male	1 h 30 min

*FH* familial hypercholesterolemia

In order to assess whether the in vitro reverted HSCs (rHSCs) have the molecular machinery both to metabolize and store vitamin A, a functional hallmark of qHSCs, we measured the retinyl ester auto-fluorescence at 328 nm by fluorescence-activated cell sorting (FACS). Approximately 70 % of the aHSCs cultured in RM are UV^+^ (Fig. 3b), and analysis of the rHSCs under UV light shows that the auto-fluorescent signal co-localizes with the intra-cytoplasmic lipid droplets, indicating that vitamin A is indeed metabolized and stored inside the lipid droplets (Fig. 3b). Although the majority of rHSCs are highly UV^+^, some of them remain UV^−^ (~30 %) (Fig 3b). To assess whether this difference in ability to store vitamin A is linked to a difference in activation status, FACS-sorted UV^+^ and UV^−^ rHSCs were analyzed for the expression of activation markers but no difference was observed (Additional file 1: Figure S3). Interestingly, the ability of RM to revert the activated phenotype is similarly shown in a HSC-derived myofibroblast population isolated from a cirrhotic patient (Additional file 1: Figure S4). To investigate whether this induced quiescent-like state is reversible, we allowed the cells to recover for 2 days in the absence or presence of transforming growth factor beta (TGFβ). In the absence of TGFβ, the cells maintain low expression levels of pro-fibrogenic genes (Fig. 3c). However, in the presence of TGFβ, the cells again upregulate the expression of *ACTA2*, *COL1A1*, and *LOX* (Fig. 3c). A viability assay was performed to rule out that the observed effects are related to cytotoxic effects of RM (Additional file 1: Figure S5A). Moreover, a 3-day recovery period with 10 % fetal bovine serum (FBS) supplemented culture medium also significantly increases the expression of *ACTA2* and *LOX* in rHSCs (Additional file 1: Figure S5B).

To evaluate whether RM can, in addition to revert, also actively prevent the culture-induced activation of HSCs, freshly isolated mouse qHSCs were seeded in normal control conditions or RM optimized for mouse HSC cultures (mRM; see “Methods”) until day 6. We find that the cells grown in mRM maintain low expression levels of *Acta2*, *Col1a1*, and *Lox*, similar to uncultured D0 mouse qHSCs and have an intermediary phenotype with large intra-cytoplasmic lipid droplets (Additional file 1: Figure S6).

### In vitro reverted human HSCs have a reduced proliferation and matrix metalloproteinase activity

**Fig. 4:**
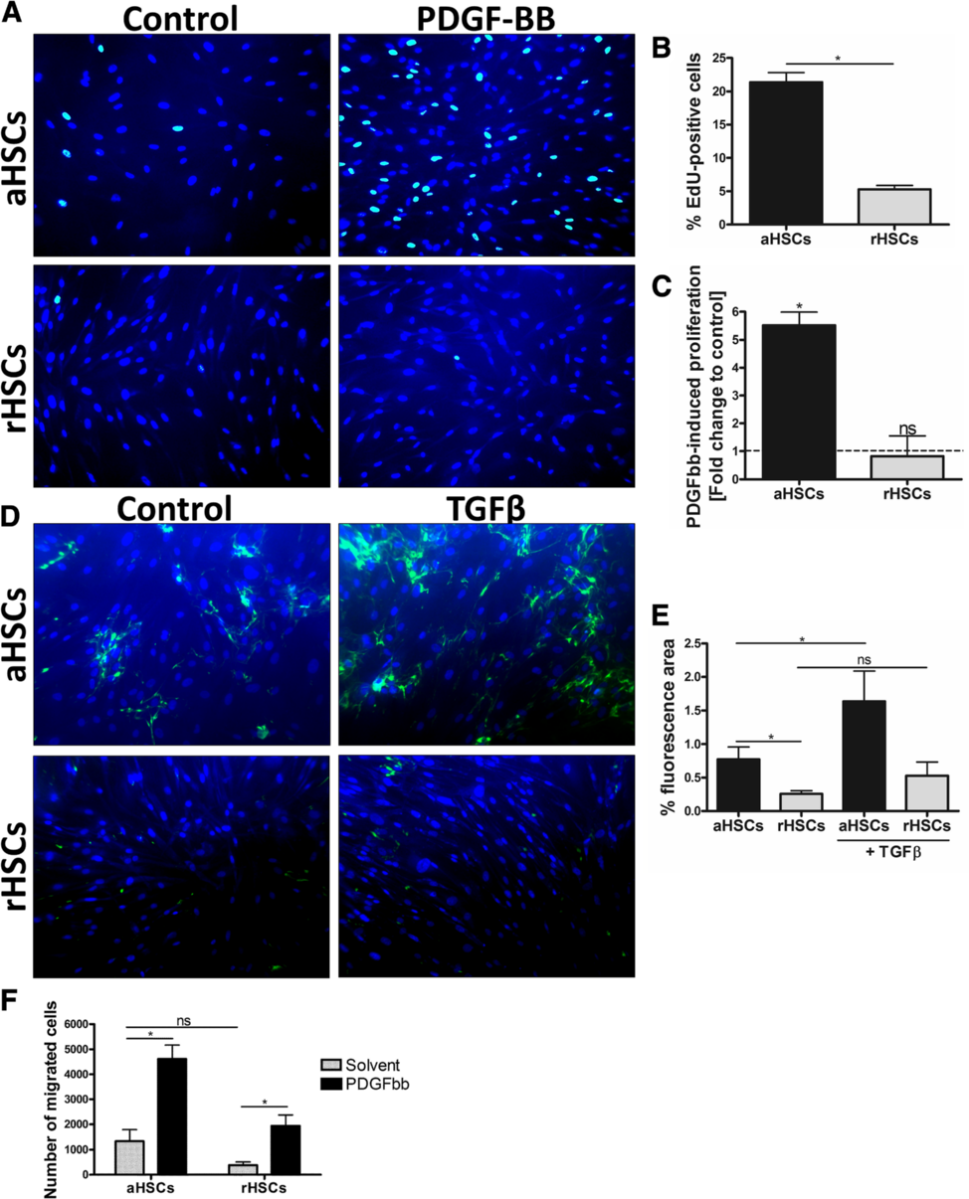
Functional comparison of human aHSCs and rHSCs. **a** EdU staining images showing the influence of RM on basal and PDGF-BB-induced HSC proliferation. **b** The percentage proliferative cells, calculated as the ratio of EdU-positive nuclei over the total (DAPI) nuclei. **c** The PDGF-BB (20 ng/mL) induced fold change in proliferation for aHSCs and rHSCs, relative to their basal condition. **d** Fluorescent images depicting the basal and TGFβ-induced (10 ng/mL) proteolytic digestion (*green*) of the fluorescein-labeled gelatin substrate by aHSCs and rHSCs. **e** The quantified basal and TGFβ-induced matrix metalloproteinase activity of aHSCs and rHSCs presented as the percentage green stained area over the total image area. **f** The PDGF-BB-inducible migration of aHSCs and rHSCs was assessed in a transwell migration assay. The aHSCs and rHSCs were seeded in collagen-coated Boyden chambers and stimulated with PDGF-BB (20 ng/mL) or with its solvent as a control, in the lower compartment. Results are presented as the number of migrated cells in each condition. The presented results are from three different donors. *ns* not significant, *p* ≥ 0.05, *p < 0.05

It is known that aHSCs functionally differ from qHSCs by their increased proliferation, unbalanced ECM homeostasis, and higher migratory potential [4]. Therefore, we further investigated whether human in vitro rHSCs functionally differ from their activated counterparts by comparing their proliferation (EdU incorporation), ECM degradation (in situ zymography), and PDGF-BB-induced migration (transwell assay) (Fig. 4). We show that RM significantly reduces the proliferation (Fig. 4a–c) and matrix metalloproteinase (MMP) activity of human aHSCs (Fig. 4d, e). Furthermore, while PDGF-BB and TGFβ increase proliferation and protease activity in aHSCs, respectively, the stimulatory effect of both growth factors is totally abrogated in rHSCs (Fig. 4a–e). Although we note a clear difference in basal, non-stimulated migration between aHSCs and rHSCs (Fig. 4f), the differences are not significant and presumably linked to the differences in proliferation. Moreover, PDGF-BB-induced migration was comparable in both groups (Fig. 4f).

### The overall gene expression profile of in vitro rHSCs is distinct from that of aHSCs and qHSCs

**Fig. 5:**
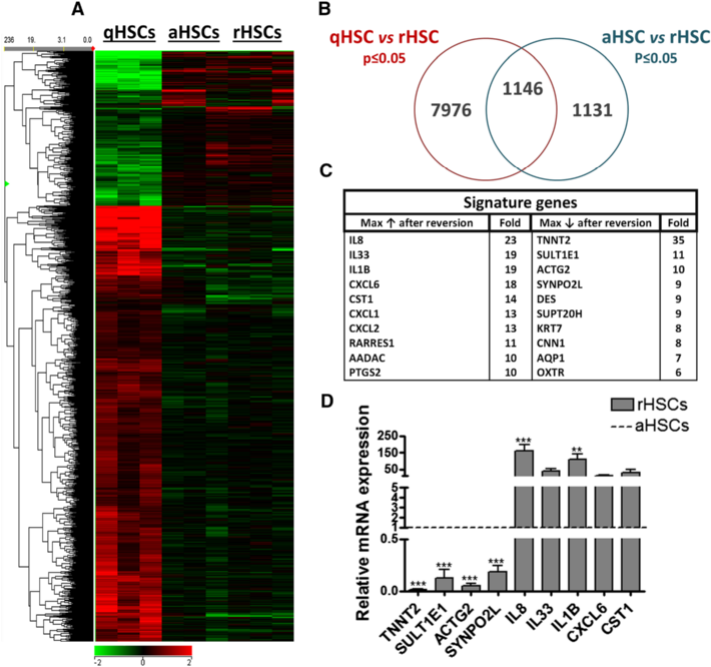
Gene expression changes elicited during the in vitro reversion of human HSC activation. **a** Heatmap representing the hierarchical clustering of genes significantly differentially regulated between aHSCs, qHSCs, and rHSCs. **b** Venn diagram showing the number of genes significantly differentially regulated between qHSCs and rHSCs or aHSCs and rHSCs. The intersection of the Venn diagram shows the number of overlapping genes significantly differentially regulated in both comparisons. **c** List of fold changes (relative to aHSCs) for the top 10 genes upregulated and downregulated in rHSCs (microarray data). **d** Confirmation of the expression levels of selected top deregulated genes by RTq-PCR. The presented results are from three different donors. **p < 0.01, ***p < 0.001

**Fig. 6:**
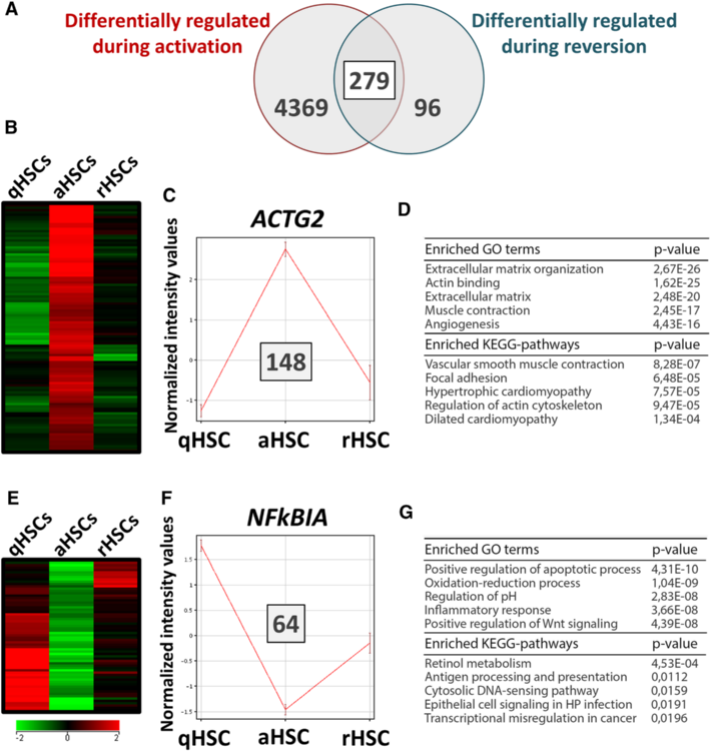
Gene expression changes during in vitro reversion of human primary HSCs inversely correlate with changes observed during in vitro HSC activation. **a** Venn diagram showing the number of genes differentially regulated during HSC activation ([qHSCs vs aHSCs], *p* ≤ 0.05 and fold change ≥2) and the number of genes differentially regulated during in vitro reversion to quiescence-like ([aHSCs vs rHSCs], *p* ≤ 0.05 and fold change ≥2). The intersection of the Venn diagram shows the number of overlapping genes between both comparisons. **b** Expression heatmap for the genes that are significantly upregulated during HSC activation and significantly downregulated during reversion to quiescence-like. **c** Profile plot showing the expression profile of *ACTG2* expression in qHSCs, aHSCs, and rHSCs as illustration for the 148 genes showing a similar expression profile. **d** The top five Gene Ontology (GO) terms and KEGG pathways associated with the set of genes represented in (**c**). **e** Expression heatmap for the genes that are significantly downregulated during HSC activation and significantly upregulated during reversion to quiescent-like cells. **f** Profile plot showing the expression profile of *NFKBIA* expression in qHSCs, aHSCs, and rHSCs as illustration for the 64 genes showing a similar expression profile. **g** The top five GO terms and KEGG pathways associated with the set of genes represented in (**f**)

To gain more insight into the gene expression changes elicited by this transient reversion into a quiescent-like state, the global gene expression profile of rHSCs was assessed and compared to that of freshly isolated, non-cultured qHSCs and culture aHSCs using the human genome U219 arrays. We find that the global gene expression profile of rHSCs resembles more closely that of aHSCs than qHSCs (Fig. 5a), with 2277 (rHSC vs aHSC) against 9122 (rHSC vs qHSC) genes expressed at significantly different levels (Student *t* test, *p* ≤ 0.05) (Fig. 5b). We find that many of the top upregulated genes in rHSCs compared to aHSCs are inflammation-related, i.e., *IL-8*, *IL-33*, *IL-1β*, *CXCL1*, *CXCL2*, and *CXCL6*, and over 60 % of the ≥2-fold deregulated genes in rHSCs are downregulated and include genes such as *TNNT2*, *SULT1E1*, and *ACTG2* (Fig. 5c, d). We were further interested in identifying potential molecular processes governing this phenotypic reversion of the activated state. We find that although a limited number of genes (*n* = 375) is at least twofold differentially regulated upon RM exposure (Fig. 6a), around 75 % (*n* = 279) of these genes are also deregulated upon activation (Fig. 6a, Additional file 2: Table S1) and 212 genes have an expression profile that negatively correlates with changes observed during HSC activation (Fig. 6b, c and Fig. 6e, f). Over 50 % of those genes are upregulated during activation and again downregulated in rHSCs (Fig. 6b, c). This set includes many well-known pro-fibrogenic genes, i.e., *COL5A1*, *ADAM12*, *LOXL1*, *ACTG2*, *NOTCH3*, and *CRYAB*, and associates with Gene Ontology (GO) terms such as “Extracellular matrix organization,” “Actin binding,” and “Muscle contraction” (Fig. 6d). Interestingly, this set of genes is enriched for different KEGG pathways of major importance in aHSCs, including “Vascular smooth muscle contraction” (*p* value 8.28 × 10^−8^), “Focal adhesion” (6.48 × 10^−5^), “Regulation of actin cytoskeleton” (9.47 × 10^−5^), and “ECM receptor interaction” (2.13 × 10^−3^) (Fig. 6d). Another 23 % of the genes show the opposite negative correlation, i.e., downregulated during activation and upregulated in rHSCs (Fig. 6e, f). This set is associated with more general GO terms and enriches for genes pertaining to “Retinol metabolism” (Fig. 6g).

### In vitro reverted human HSCs upregulate the expression of in vivo inactivated-HSC-specific markers

To determine whether human HSCs reverted to quiescence-like by RM share characteristics with in vivo inactivated HSCs (iaHSCs) found in the mouse liver after recovery from experimentally induced fibrosis [13, 14], we measured the expression of a panel of genes (*n* = 10) previously identified as upregulated or specific for in vivo iaHSCs compared to aHSCs and qHSCs [13, 15], in aHSCs and *r*HSCs from three HSC donors. Although there was inter-donor variation, we find all 10 genes to be consistently upregulated in rHSCs (Table 2).

**Table 2:** Overview of the fold difference in mRNA expression levels of in vivo iaHSC signature genes in rHSCs and aHSCs

		Fold change rHSC vs aHSC		
Gene	Accession	Donor L4	Donor L8	Donor L12
*CXCL1*	NM_001511	52	30.7	26
*CXCL2*	NM_002089	39.1	7.3	23.6
*CXCL10*	NM_001565	3.7	32.7	nd
*CTSS *(tv2)	NM_001199739	27.6	20.5	10.1
*LY86*	NM_004271	1.8	26.4	6.2
*CAPN6*	NM_014289	2.6	5.2	14.1
*RND1*	NM_014470	1.9	11.7	6.6
*KRT20*	NM_019010	6	nd	3.9
*CX3CR1* (tv1)	NM_001171174	3.2	nd	5.4
*GPC3* (tv4)	NM_001164619	1.5	1.8	1.2

tv transcript variant, nd not detected

## Discussion

A strong body of evidence suggests that upon removal of the etiological agent, liver fibrosis can spontaneously revert in patients with secondary biliary fibrosis [20], hepatitis B [21] and C [22], non-alcoholic steatohepatitis [23], and autoimmune hepatitis [24]. Recent studies in rodent models of liver fibrosis have convincingly shown that fibrosis regression is in part driven by the reversion of aHSCs to quiescence-like HSCs [13, 14]. While this ability to revert was shown both in vitro [11, 12] and in vivo [13, 14] for rodent HSCs, it remains unknown whether the activated phenotype of human HSCs can be reverted as well. Moreover, the molecular mechanisms underlying the intermediate phenotype of reverted HSCs remain largely unknown. Therefore, our study aimed at identifying conditions that can revert the activated status of human HSCs and to map the molecular events associated with this phenotype reversion.

Our experiments reveal a role for EGF and FGF2 in negatively regulating the activated phenotype of human HSCs. The identification of EGF as a negative regulator of HSC activation is counterintuitive. Indeed, EGF is overexpressed in the fibrotic liver [25], plays a central role during liver regeneration [26], and is member of a 186-gene signature predictive of progressive cirrhosis, hepatocellular carcinoma, and death in patients with cirrhosis [27]. Moreover, the pharmacological inhibition of the EGF receptor was shown to attenuate liver fibrosis [28]. However, to our knowledge, the present study is the first one to address the direct effect of EGF stimulation on the expression of activation markers in human primary HSCs. We find that FGF2, another growth factor known to be overexpressed in the fibrotic liver [29], synergized the negative regulation of *ACTA2* and *LOX* expression by EGF. Although this synergistic effect was not very pronounced, it was consistent in HSCs isolated from patients with different backgrounds (i.e., age and gender). A synergy between EGF and FGF2 in the negative regulation of human HSC activation has not been shown yet; however, a previous study already showed that EGF and FGF2 can act synergistically to increase the proliferation of neural precursor cells [30]. Moreover, this same study showed that FGF2 can induce EGF responsiveness, providing a possible explanation for our observation. We also find that a mix of palmitic acid and retinol induces the formation of retinyl ester-containing intra-cytoplasmic lipid droplets in human HSCs. This is in concordance with previous work by Lee et al. showing that palmitate induces the upregulation of ADRP, a lipid droplet associated protein, and that this effect was potentiated by retinol in human HSCs [31]. We find that adding oleic acid further increased the number of lipid droplets and lipid droplet-positive cells induced by retinol and palmitic acid (data not shown). Interestingly, although the combination of oleic acid, palmitic acid, and retinol only has a subtle effect on the expression of activation markers, it strongly potentiates the effect of EGF and FGF2 on the reduced expression of *ACTA2*, *COL1A1*, and *LOX*. Although not all cells incorporated lipid droplets and around 30 % of the cells remained UV^−^, no difference was found in the overall expression levels of the different activation markers between UV^+^ and UV^−^ rHSCs. This indicates that HSCs are heterogeneous in their capacity for retinoid and lipid storage, corroborating previous studies by D’Ambrosio et al. [19]. Furthermore, it suggests that the potentiated effect observed for RM is not dependent on the presence of retinyl ester-containing lipid droplets.

Although our results indicate the ability of RM to revert the activated phenotype of HSCs isolated from a cirrhotic patient, the therapeutic relevance of this finding remains to be demonstrated. Indeed, the experimental use of in vivo activated human HSCs necessitates their culture expansion, during which the cells are exposed to the same artificial environment as the in vitro activated HSCs used in the present study. Although not shown for human HSCs yet, we recently demonstrated that the gene expression profile of mouse HSCs is significantly altered as soon as 10 h after in vitro culture [32, 33]. Therefore, we believe that even a relatively short-term culture expansion of in vivo aHSCs will result in a deregulated gene expression profile that is not necessarily representative of the in vivo situation anymore. In addition to a reversion of the activated phenotype, we show that RM can prevent the culture-induced activation of qHSCs. Because human qHSC isolations are characterized by modest yields and freshly isolated cells are culture expanded during which they quickly acquire an activated phenotype, these experiments were performed on freshly isolated mouse qHSCs.

Microarray analysis of non-cultured human qHSCs and in vitro aHSCs and rHSCs reveals that the global gene expression profile of rHSCs was still very similar to that of aHSCs, indicating that this reversal in phenotype is not substantiated by a thorough reprogramming at the transcriptional level. Indeed, our study found only 375 genes to be differentially regulated between rHSCs and aHSCs. Although this corresponds to only 1.8 % of all the genes represented on the HGU219 array, ~75 % of these genes are also differentially regulated during activation and over 56 % show an expression profile that negatively correlates during activation and reversion. The major part of those genes is upregulated and again downregulated during activation and reversion, respectively, and is enriched for genes that are involved in pathways that are known to play major roles in aHSCs such as “Vascular smooth muscle contraction” and “Focal adhesion”. Therefore, changes in expression of these genes probably underpin the observed shift in HSC phenotype. Interestingly, serum response factor (SRF) and specificity protein 1 (SP1) were predicted to be the transcription factors with the highest importance and occurrence score, respectively, for the binding of common regulatory elements for this set of genes (http://dire.dcode.org/). SRF is mainly involved in cell differentiation by regulating genes that control cell growth, cytoskeletal organization, contractility, and motility [34]. In rodent HSCs, Srf expression was shown to be inducible by TGFβ and small interfering RNA (siRNA)-mediated knockdown of Srf resulted in the downregulation of *Acta2* expression [35]. SP1, a member of the Krüppel-like family that was shown to have an increased DNA binding activity in aHSCs, co-operates with Krüppel-like factor 6 (KLF6) to induce the expression of *TGFβ* and urokinase plasminogen activator (*uPA*), which promotes the activation of latent TGFβ [36, 37]. Other pro-fibrogenic genes regulated by SP1 include *Col1a2* and *Timp1* [38, 39]. In rodent models of kidney, lung, and liver fibrosis, SP1 targeting was shown to be a powerful anti-fibrotic strategy [40]. This anti-fibrotic effect is presumably due to the abrogation of SP1 activity in myofibroblasts, as it was shown that SP1 targeting inhibits the expression of pro-fibrogenic genes as well as proliferation in rat aHSCs [41]. Future research will show whether targeting of both these transcription factors in fibrotic patients might represent a therapeutic strategy to revert the activated phenotype of human HSCs.

In inverse analogy to the differentiation of adipocytes from pre-adipocytes, anti-adipogenic regulation has been shown to underlie HSC activation [42]. Because both oleic acid and palmitic acid are reported to stimulate adipocytic differentiation [43], we speculate that they potentiate the negative effect of EGF and FGF2 on the expression of activation markers by enforcing the transdifferentiation of human aHSCs towards the more adipogenic qHSC phenotype. The loss of PPARγ activity, the master regulator of adipogenesis, has been found to play an important role during HSC activation [44–46], and overexpression in activated HSCs has been shown to induce a phenotypic switch to a more quiescent-like phenotype [11]. Surprisingly, the adipogenic phenotype of rHSCs was not supported by an increased expression of *PPARγ* and its downstream effector genes. Recent studies show that *PPARγ* induction is a multistep process, necessitating both epigenetic reconfiguration [47, 48] and chromatin remodeling [49]. Furthermore, transcriptional activities of PPARγ have been shown to require chromatin presetting [50], and in turn, PPARγ can lead to post-translational histone modifications (for review, see [51]) to further drive and maintain the adipogenic transcription program. Seemingly, the adipogenic cue provided by RM or the treatment duration is an insufficient trigger for this multistep process to take place in activated primary human HSCs. Nevertheless, we find that RM induces the expression of ADRP (Additional file 1: Figure S7), a protein known to be expressed early during adipogenesis and previously shown to be sufficient to stimulate lipid droplet formation and accumulation independently of PPARγ or other lipogenic genes [52]. Moreover, work by Lee et al. previously demonstrated a role for palmitic acid and retinol in upregulating the expression of ADRP [53] in LX-2 cells, an immortalized human HSC line. The authors report that the treatment correlated with reduced *ACTA2* and *COL1A1* expression and ADRP knockdown abrogated the treatment’s effect [53]. In contrast, our experiments do not reveal a direct role for ADRP in the negative regulation of activation genes. Nevertheless, increased *ADRP* expression provides a possible explanation for the potentiating effect of the dietary supplements on the downregulation of *ACTA2*, *COL1A1*, and *LOX* by EGF and FGF2.

At the functional level, we show the RM’s potency to not only reduce basal proliferation and MMP activity of the cells, but also to actively counteract the increased functionality of HSCs by well-known pro-fibrogenic factors. The mechanism underlying these effects however remains unknown and needs further attention.

Recently, Kisseleva et al. for the first time described the unique phenotypic characteristics of murine in vivo iaHSCs and identified a set of genes highly expressed in iaHSCs compared to qHSCs and/or aHSCs [13, 15]. Interestingly, we find that these genes were consistently upregulated in rHSCs, derived from different donors. Although the relevance of this observation remains to be demonstrated, their increased expression in in vitro reverted human HSCs suggests also a potential role for these genes in promoting a quiescent-like phenotype in human HSCs.

## Conclusions

In conclusion, this study for the first time provides evidence that human HSCs can be reverted to a quiescent-like state and provides a first phenotypic and genomic characterization of in vitro reverted human HSCs.

## Methods

### Patient samples

The protocol and experiments were approved by the ethical committees of St-Luc Hospital and the Faculty of Medicine of Université Catholique de Louvain. An agreement from the Belgian Ministry of Health was obtained for the Hepatocytes and Hepatic Stem Cells Bank. A written and signed informed consent has been obtained for each human liver used in the current study. Five livers, for which the clinical characteristics are summarized in Table 1, were used in the current study. Additionally, commercially available primary human liver myofibroblasts derived essentially from hepatic stellate cells and portal fibroblasts (Tebu-Bio NV, Antwerp, Belgium) was used.

### Isolation and in vitro reversion of human activated HSCs

Human aHSCs were obtained by plating the qHSC-enriched population obtained after Nycodenz (Myegaard, Oslo, Norway) gradient centrifugation of the non-parenchymal cell fraction [54]. Homogeneous populations of aHSCs were obtained after three passages in Dulbecco’s modified Eagle’s medium (DMEM) supplemented with 10 % FBS at 37 °C in a humidified atmosphere with 5 % CO_2_ and subsequently cultured in 1 % FBS supplemented medium. Prior to each passage, cells were washed with phosphate buffered saline (PBS) and lifted using 0.05 % trypsin (Lonza). For reversion of the activated phenotype, human aHSCs were seeded at a density of 10,000 cells/cm^2^ (unless stated differently), and 24 h later, the cells were washed and incubated with DMEM supplemented with 1 % FBS, 20 ng/mL epidermal growth factor (EGF) (Peprotech, London, UK), 10 ng/mL fibroblast growth factor 2 (FGF2) (Peprotech), 100 μM oleic acid (Sigma), 100 μM palmitic acid (Sigma), and 5 μM retinol (Sigma). The medium was refreshed every 2 days for 5 days, and the cells were harvested for further analysis on day 6.

### Isolation of non-plated human quiescent HSCs

The human liver parenchymal and non-parenchymal cell fractions were separated from each other by sequential perfusion of liver pieces with pre-warmed EGTA-containing EBSS medium (Lonza, Verviers, Belgium) and a digestion enzyme solution (EBSS supplemented with 0.9 mg/mL collagenase P and 0.03 mg/mL soybean trypsin inhibitor (Roche)) for 9 to 12 min. Collagenase digestion was stopped with ice-cold M199 wash medium (Lonza) containing 0.03 mg/mL of soybean trypsin inhibitor and 100 mL/L of human plasma [55]. After filtration, the non-parenchymal cells were separated from the parenchymal cells by subsequent low-speed centrifugation steps (50*g*) and submitted to an additional centrifugation step (640*g*). Non-parenchymal cell pellets were then resuspended and cryopreserved in DMEM (Lonza) supplemented with 20 % FBS (Biochrom GmbH, Berlin, Germany) and 5 % dimethyl sulfoxide (DMSO) (Sigma, St. Louis, MO, USA). The isolation of high-purity human qHSCs was performed as described previously [56]. In brief, dissociated and washed single non-parenchymal cells were suspended in a 5 % FBS, 2mM EDTA (Sigma) buffer and incubated for 30 min at 4 °C with anti-CD32 (Abcam, Cambridge, UK) and anti-CD45 (BD Biosciences, San Jose, CA, USA). 7-Aminoactinomycin (7-AAD) (eBioscience, San Diego, CA, USA) was used for the exclusion of non-viable cells. Pure populations of qHSCs were sorted as CD32^−^CD45^−^UV^+^ cells, using FACSAria™ (BD Biosciences). RNA from freshly isolated qHSCs was obtained using RNeasy Micro Kit (Qiagen). RNA samples were amplified using the Ovation Pico WTA system V2 (NuGEN).

Mouse HSCs were isolated from male BalbC mice (age 20–25 weeks) (Charles River Laboratories, L’arbresle, France) and cultured in DMEM with 10 % FBS, as described previously [57]. For treatment experiments with mouse HSC reverting medium (mRM) (DMEM supplemented with 10 % FBS, 40 ng/mL EGF, 20 ng/mL FGF2, 100 μM oleic acid, 100 μM palmitic acid, and 5 μM retinol), freshly isolated mouse qHSCs were seeded at a density of 7.500 cells/cm^2^. The inhibition of activation was assessed by relative comparison to control cells cultured in DMEM supplemented with 10 % FBS for the same period of time. All procedures on animals were carried out in accordance with the University’s guidelines for the care and use of laboratory animals in research. The performed experiments were approved by the ethical committee of the Vrije Universiteit Brussel in project 12-212-1.

### Gene expression profiling and analysis

Total RNA originating from cultured (activated and reverted HSCs; *n* = 3 from corresponding donors) and uncultured (FACS-sorted quiescent HSCs; *n* = 3 from two corresponding donors) primary human HSCs was isolated by using RNeasy Micro Kit (Qiagen GmbH, Hilden, Germany) following the manufacturer’s recommendations. Total RNA concentration and quality control was assessed using RNA 6000 Pico Kit (Agilent, Santa Clara, CA, USA). RNA samples were amplified by using Ovation PicoSL System V2 (NuGene Technologies, CA, USA) and ENCORe Biotin module (NuGene Technologies). Amplified and purified RNA samples were labeled and hybridized to the Affymetrix HG-U219 GeneChip (Affymetrix, Santa Clara, California, USA). Data normalization and analysis was performed using GeneSpring GX12 (Agilent, Santa Clara, CA, USA) as described previously [32]. Briefly, Affymetrix gene expression data were normalized using the robust multi-array algorithm [58]. For the detection of differentially expressed genes, a *p* value cutoff of 0.05 was used in combination with a fold change cutoff of 2.0. Functional analysis of gene expression data (gene ontology (GO) and Kyoto Encyclopedia of Genes and Genomes (KEGG) pathways) was conducted using an open access, high-level cross-platform microarray dataset analysis tool (InCroMAP) (http://www.ra.cs.uni-tuebingen.de/software/InCroMAP/). Raw data are made publically available on the NCBI Gene Expression Omnibus database, with accession number GSE68001.

### Immunocytochemistry

Nycodenz-isolated HSCs cultured for 1 day or for three passages were washed with PBS and fixed for 10 min with 4 % buffered formaldehyde (Merck, Darmstadt, Germany). Following permeabilization with 0.1 % Triton X-100 (in PBS containing 1 % bovine serum albumin), cells were incubated overnight with anti-αSMA (1/1000) (Sigma). Primary antibody binding was visualized using an Alexa488-labeled secondary antibody (1/200) (Invitrogen, Eugene, OR, USA). Images were taken with an AxioCam MRc5 digital camera (Carl Zeiss). NCAM1, nestin, and desmin stainings were performed as described previously [59]. Briefly, the cells were fixed, endogenous peroxidase was eliminated, and cells were permeabilized using PBS containing 1 % Triton X-100 (Sigma). Thereafter, the cells were blocked by 1-h incubation in PBS containing 1 % bovine serum albumin (Sigma), incubated with primary anti-NCAM1 (1/100) (Abcam), anti-Nestin (1/1000) (Abcam), or anti-Desmin (1/50) (Abcam) for 1 h, washed and incubated with secondary antibody (Envision, Dako) for 30 min. Detection was performed after 5 min incubation with liquid DAB and substrate chromogen (Dako). Counterstaining was performed using Mayer’s hematoxylin for 10 min. Preparations were then mounted for microscopic analysis (DMIL, Leica, Belgium).

### Lipid staining

The cells were washed with PBS and fixed in 10 % formalin solution. The cells were then washed with deionized water, incubated with isopropanol for 5 min, and stained with diluted (3/2 in water) and filtered Oil Red O (0.3 % (*w*/*v*) in 99 % isopropanol) (Sigma) for 20 min. The red-stained lipid droplets were visualized with light microscopy (Carl Zeiss).

### Western blot

Cells were washed with ice-cold PBS and scraped with ice-cold lysis buffer (170 mM NaCl, 10 mM EDTA, 50 mM Tris pH 7.4, 50 mM NaF, 0.2 mM dithiothreitol, and 0.5 % NP-40) supplemented with protease (Roche Diagnostics, Mannheim, Germany) and phosphatase (Roche Diagnostics) inhibitors. Protein concentrations were determined using the BCA protein assay kit (Pierce Chemical Co, Rockford, IL, USA). Fifteen microgram of protein was separated on a 8 % Tris-glycine SDS-polyacrylamide gel and electroblotted onto polyvinylidene difluoride membranes (Amersham Biosciences, Little Chalfront, UK) using a wet blotting apparatus (Mini Trans-Blot Cell, BioRad, Nazareth, Belgium). Blots were blocked with 5 % milk powder in Tris buffered saline (TBS) with 0.2 % Tween (Sigma) and subsequently incubated overnight with primary anti-PDGFRβ (diluted 1/1000 in blocking buffer) (Abcam), anti-αSMA (1/50) (Dako), anti-COL1A1 (1/1000) (Abcam), or anti-GAPDH (diluted 1/30,000) (Abcam). The membranes were washed and incubated with a horseradish peroxidase-conjugated secondary antibody (1/20,000) (Dako, Glostrup, Denmark) for 1 h, and the antigen was visualized by enhanced chemiluminescence using ECL substrate (Pierce Chemical Co.).

### Proliferation assay

Cell proliferation of human HSCs was assessed with the Click-iT EdU Cell Proliferation Assay Kit (Invitrogen, Eugene, OR, USA). HSCs were cultured under control conditions or in RM for 6 days. On day 6, the cells were labeled with 10 μM EdU for 6 h and subsequently formalin fixed and mounted with Prolong Gold antifade reagent with DAPI (Invitrogen). EdU incorporation was visualized according to the manufacturer’s instructions. The percentage proliferation was calculated as the ratio of EdU-positive cells to DAPI-positive cells. For the PDGF-BB stimulation experiments, the control medium and RM were supplemented with 20 ng/mL human recombinant PDGF-BB for 48 h prior to EdU labeling. The quantification was performed on at least 500 cells per donor and is presented as the mean percentage measured in three different donors.

### In situ zymography

The matrix metalloproteinase activity of human HSCs was assessed using the highly quenched, fluorescein-labeled pig skin gelatin (DQ™ gelatin) (Invitrogen). Upon proteolytic digestion, its green fluorescence is revealed and can be used to measure enzymatic activity. A 1 mg/mL stock solution of DQ gelatin was prepared using deionized water and stored at 4 °C. Prior to cell seeding (20,000 cells/cm^2^), glass coverslips (12 mm diameter) were coated with 50 μg DQ gelatin for 1 h. Twenty-four hours post-seeding, the cells were washed with serum-free DMEM and cultured for 6 days under control conditions or RM. On day 6, the cells were formalin fixed and mounted with Prolong Gold antifade reagent with DAPI. The percentage green stained area was calculated using ImageJ (http://imagej.nih.gov/ij/index.html). For the TGFβ stimulation experiments, the control medium and RM were supplemented with 10 ng/mL human recombinant TGFβ for 48 h prior to being fixed and analyzed. The quantification was performed on 5–10 images per donor and is presented as the mean percentage measured in five different donors.

### Migration assay

Both aHSCs and rHSCs were seeded in collagen-coated Boyden chambers (Millipore) (40,000 cells/chamber) in serum-free DMEM. After 60 min, the chambers were transferred to wells with 20 ng/mL PDGF-BB (R&D Systems, Minneapolis, MN, USA) or its solvent as a control. After 16 h, non-migrated cells were cleared and migrated cells were fixed with ice-cold 100 % methanol and mounted with Prolong Gold antifade reagent with DAPI. The quantification was performed by manually counting the totality of migrated cells on images covering the entire membrane and is represented as the mean of three different donors.

### Viability/cytotoxicity assay

For the LIVE/DEAD viability/cytotoxicity assay (Life Technologies), aHSCs and rHSCs were incubated with PBS containing 4 μM ethidium homodimer and 2 μM calcein for 15 min at 37 °C, washed thoroughly with PBS, and subsequently analyzed under a fluorescent microscope (Carl Zeiss). Images were taken with an AxioCam MRc5 digital camera (Carl Zeiss).

### RNA purification and RTq-PCR

RNA was extracted and purified from cultured and uncultured cells using the ReliaPrep RNA Cell Miniprep System (Promega, Madison, WI, USA). Total RNA was converted to cDNA by reverse transcription using the Revert Aid Kit (Thermo Fisher Scientific, St. Leon-Rot, Germany). Quantitative real-time polymerase chain reaction was performed using the GoTaq qPCR Master Mix with BRYTE green (Promega). A 7500 real-time PCR system was used and data was analyzed using System SDS software v2.0.6 (Applied Biosystems). Fold change differences between samples were determined using the comparative Ct method (∆∆Ct). The expression level of different target genes, relative to glyceraldehyde-3-phosphate dehydrogenase (*GAPDH*) and the calibrator, was given by 2-∆∆Ct. Gene-specific primers were produced by Integrated DNA Technologies (Leuven, Belgium).

### Statistical analysis

GraphPad Prism v4.0.0 (GraphPad Software, La Jolla, CA, USA) was used for statistical analysis. Data in the figures are expressed as means ± SEM. Differences among groups were tested for statistical significance by Student *t* test or analysis of variance (ANOVA) followed by Tukey’s test, depending on the number of groups (ns = not significant, *p* ≤ 0.05, *p < 0.05, **p < 0.01, ***p < 0.001).

## Additional files

Additional file 1: 
**Supplementary figures.** (ZIP 4596 kb) Additional file 2: 
**List of overlapping genes at least 2-fold differentially expressed upon** ***in vitro*** **activation and reversion of human HSCs.** (XLSX 27 kb)

## Abbreviations

ECM: extracellular matrix
EGF: epidermal growth factor
FGF2: fibroblast growth factor 2
GO: Gene Ontology
HSC: hepatic stellate cell—activated (a), quiescent (q), reverted (r), inactivated (ia)
KEGG: Kyoto Encyclopedia of Genes and Genomes
OA: oleic acid
PA: palmitic acid
R: retinol
RM: reverting medium
UV: ultraviolet
